# Equal Opportunity Interference: Both L1 and L2 Influence L3 Morpho-Syntactic Processing

**DOI:** 10.3389/fpsyg.2021.673535

**Published:** 2021-05-28

**Authors:** Nawras Abbas, Tamar Degani, Anat Prior

**Affiliations:** ^1^Department of Learning Disabilities, University of Haifa, Haifa, Israel; ^2^Department of Communication Sciences and Disorders, University of Haifa, Haifa, Israel; ^3^Edmond J. Safra Brain Research Center for Learning Disabilities, University of Haifa, Haifa, Israel

**Keywords:** trilingualism, cross-language influence, morphosyntax, English as a foreign language, interference

## Abstract

We investigated cross-language influences from the first (L1) and second (L2) languages in third (L3) language processing, to examine how order of acquisition and proficiency modulate the degree of cross-language influences, and whether these cross-language influences manifest differently in online and offline measures of L3 processing. The study focused on morpho-syntactic processing of English as an L3 among Arabic-Hebrew-English university student trilinguals (*n* = 44). Importantly, both L1 (Arabic) and L2 (Hebrew) of participants are typologically distant from L3 (English), which allows overcoming confounds of previous research. Performance of trilinguals was compared to that of native English monolingual controls (*n* = 37). To investigate the source of cross-language influences, critical stimuli were ungrammatical sentences in English, which when translated could be grammatical in L1, in L2 or in both. Thus, the L3 morpho-syntactic structures included in the study were a mismatch with L1, a mismatch with L2, a Double mismatch, with both L1 and L2, or a no mismatch condition. Participants read the English sentences while their eye-movements were recorded (online measure), and they also performed grammaticality judgments following each sentence (offline measure). Across both measures, cross-language influences were assessed by comparing the performance of the trilinguals in each of the critical interference conditions to the no-interference condition, and by comparing their performance to that of the monolingual controls. L1 interference was evident in first pass sentence reading, and marginally in offline grammaticality judgment, and L2 interference was robust across second pass reading and grammaticality judgments. These results suggest that either L1 or the L2 can be the source of cross-language influences in L3 processing, but with different time-courses. The findings highlight the difference between online and offline measures of performance: processing language in real-time reflects mainly automatic activation of morpho-syntactic structures, whereas offline judgments might also involve strategic and meta-linguistic decision making. Together, the findings show that during L3 processing, trilinguals have access to all previously acquired linguistic knowledge, and that the multilingual language system is fully interactive.

## Introduction

Multilingualism can be considered a conventional feature of linguistic experience and maturity (Hammarberg, 2010). The growing prevalence of third language (L3) acquisition raises important theoretical considerations of how an additional language is represented and processed by multilingual speakers (Slabakova, 2017). While in second language (L2) acquisition learners rely solely on their experience with one language, in L3 acquisition two pre-existing systems of linguistic representations are available (Westergaard, 2019; Puig-Mayenco et al., 2020). Thus, investigating L3 acquisition allows researchers to clarify specific factors that might be confounded in L1 or L2 acquisition, such as how proficiency in a previous language might influence acquiring an additional language (Flynn et al., 2004).

Cross language influence is evident when acquisition or processing of one language is influenced by existing knowledge of other languages (Cenoz, 2001). Such influences can be facilitative, when structures of two languages are similar (positive transfer), but can also lead to language interference (negative transfer), in the presence of structural differences between the languages in question (Isurin, 2005; MacWhinney, 2005). There is a sizeable body of knowledge regarding how L1 can influence L2 processing (Hopp, 2010; Prior et al., 2017) and vice versa (Dussias and Sagarra, 2007; Degani et al., 2011), but our current understanding of how linguistic knowledge in L1 and/or L2 influences L3 learning and processing is far from being complete (Angelovska and Hahn, 2012; Rothman et al., 2019; Lago et al., 2020; Puig-Mayenco et al., 2020).

### Modulating Factors of Cross-Language Influences

One important dimension that has been emphasized as impacting cross-language influences in L3 learning and processing is the order of acquisition and/or proficiency in each of the background languages (Williams and Hammarberg, 2009). In addition, the typological similarity between each of the background languages and the L3 (Rothman and Halloran, 2013; Rothman, 2015) has also been identified as an important factor determining CLI in L3. Crucially, in much previous research these variables have been confounded or have been pitted against each other (e.g., Giancaspro et al., 2015; Puig-Mayenco et al., 2020). In the current study, we examine cross-language influence as a function of order of acquisition and/or proficiency in each of the background languages, irrespective of typological similarity, because for the examined population both L1 (Arabic) and L2 (Hebrew) are similarly typologically distant from the target L3 (English). Importantly, by using eye tracking as a measure of comprehension, we also examine the time-course of cross-language influences from each of the background languages, an issue which has received only very little attention in the extant literature.

When considering L3 processing, both L1 and L2 are potential sources of cross-language influences. However, there is ongoing debate regarding how these influences may play out, and whether one of the background languages becomes the “default supplier” of cross-language influence (L1/L2) in L3 use. A strong preference for one of the previously acquired languages as providing cross-language influences for L3 has been suggested in some cases. For instance, some studies have identified L1 as the main source of cross-language influences in the acquisition of L3 in syntax and in lexicon (e.g., Gollan et al., 2002; Angelovska and Hahn, 2012). Hermas (2010) reported that among Arabic native speakers with L2 French and L3 English, the initial state of L3 syntax acquisition was influenced exclusively by the L1. Similarly, Lindqvist (2009) found that L1 was the main source of lexical influence on L3 French, among three groups with different background language combinations.

In contrast, many L3 acquisition studies have also identified cross-language influences that originate in the learner’s L2 (e.g., Ringbom, 1987; Hammarberg, 2001; Bardel and Falk, 2007; Fallah et al., 2016). The “L2 Status Factor” theory explained that learners tend to activate the L2, rather than the L1, in L3 acquisition, because L2 is more similar to L3 with respect to the learning situation, age of onset, and degree of metalinguistic knowledge (Bardel and Falk, 2007; Falk and Bardel, 2011). Additionally, Bardel and Falk (2012), following neurolinguistic claims (Ullman, 2005), suggested that both L2 and L3 as non-native languages are stored in declarative memory, while the native language is stored in procedural memory. A study by Falk and Bardel (2011) in the domain of syntax, supported this hypothesis, by demonstrating that L2 superseded L1 as a source of both facilitation and interference in the L3. Specifically, using grammaticality judgment and a correction task, the study examined the placement of object pronouns in L3 German among two groups; L1 French-L2 English, and L1 English-L2 French. The results indicated that grammaticality judgments were influenced by participants’ L2, and not L1, in both groups, suggesting that L2 had a stronger role than L1 in L3 acquisition (see also Angelovska and Hahn, 2012).

Lastly, recent models question the role of order of acquisition in granting privileged status to either L1 or L2 in cross-language influences on L3. For example, the *Scalpel Model* (Slabakova, 2017) argues against wholesale cross-language influence of previously acquired languages at the initial stages of acquisition, and instead posits that cross-language influences can come from the L1 or the L2 or both. Similarly, the *Linguistic Proximity Model* (Westergaard et al., 2017; Westergaard, 2019) suggests that in L3 acquisition, learners have access to all previously acquired languages, and that language acquisition is cumulative. In support of this claim, Westergaard et al. (2017) demonstrated that in a grammaticality judgment task in English (studied as a foreign language), monolingual Norwegian speaking children over accepted ungrammatical sentences, whereas Russian-Norwegian bilingual children and monolingual Russian speaking children noticed significantly more errors. However, the bilinguals scored lower than the L1 Russian speakers on grammatical sentences, suggesting the presence of interference from Norwegian. These results support the hypothesis that both previously acquired languages remain active and influence subsequent L3 acquisition, and that cross-language influences can be either facilitative or non-facilitative.

Several studies have found that either L1, L2, or both may contribute to cross-language influences in L3 acquisition (e.g., Flynn et al., 2004). For example, Bruhn de Garavito and Perpiñán (2014) found that speakers of L1 French – L2 English, at the initial stages of learning Spanish L3, rely in some situations on their L1 French grammar to interpret facts, and in other situations, they rely on their L2 English grammar. These findings suggest that L1 and L2 were both available and used whenever they facilitate processing of the input.

In addition to order of acquisition, individuals’ proficiency in each of the background languages has also been cited as possibly contributing to the strength of L1 or L2 as sources of cross-language influences on L3. Specifically, high proficiency in a background language enables it to be influential in the acquisition of a new language (Williams and Hammarberg, 2009). For example, German was identified as the strongest source of cross-linguistic influence in acquisition of English, for monolingual German speaking adolescents but also for heritage speakers (of Turkish or Russian) who were immersed in German at the time of testing (Lorenz et al., 2019).

Finally, language typology has also been suggested as an important and influential factor in determining cross-language influences. The assumption is that the language that is more typologically similar to the L3, whether it is the L1 or the L2, will provide stronger influence during L3 acquisition and processing (Falk and Bardel, 2010; Angelovska and Hahn, 2012), as described by The *Typological Primacy Model* (Rothman, 2010, 2011, 2015).

The role of typology has been demonstrated in several studies. For example, Giancaspro et al. (2015) found that speakers of English and Spanish were dominantly influence by Spanish when learning L3 Brazilian Portuguese, regardless of whether Spanish was their L1 or L2. Analogous findings, of stronger influence from the typologically closer language, have also been demonstrated in the lexical domain (e.g., Ringbom, 1987; Poarch and Van Hell, 2014).

Importantly, studies that investigate the interplay of these various factors are often limited by confounds among them (Ecke, 2015; Rothman, 2015). Thus, many studies on L3 processing investigated the use of an L3 after the acquisition of an L2 which is more similar to L3 than is the L1 (e.g., De Angelis and Selinker, 2001). In such studies the effects of order of acquisition cannot be separated from those of typological similarity (Ecke, 2015). A few studies have tried to disentangle such combined effects, but provided mixed results. Cenoz (2001), for example, investigated bilingual speakers of Spanish and Basque learning English as an L3. Spanish is typologically more similar to English than Basque, but the results showed greater cross-language influences from Spanish when learning L3 English only when Spanish was the learner’s L2, not when it was the L1. This finding demonstrated that beyond language typology, the L2 has an additive effect on cross-linguistic influence (see also Bardel and Falk, 2007, for similar results). On the other hand, two studies by Singleton and O’Laoire (2004, 2005) demonstrated that in the lexical domain the typology factor was stronger than the L2 status factor. Specifically, English, which is typologically closer to French in lexical terms, was the dominant source of cross-language influences in learning French as an L3, both for English-L1– Irish-L2 bilinguals, and for bilinguals with both Irish and English as their L1s.

The results described above emphasize the difficulty of investigating cross-language influence in L3 processing, and the unique challenge of separating the impact of various factors. Thus, studies that have directly contrasted typology and order of acquisition as determining factors for cross-language influence have not reached a coherent conclusion – with some results identifying typology as the critical factor, and others identifying order of acquisition. The current study was designed to further investigate this issue, in a design that effectively neutralizes the typological factor, by studying L3 processing in trilinguals for whom both L1 and L2 are typologically *distant* from the L3.

### The Current Study

The main goal of this study is to examine whether L1 or L2 can be identified as an exclusive source of cross-language influence, or whether the entire linguistic repertoire is activated when processing L3 morpho-syntax. The specific population and methods we adopted allow us to complement previous research in several important ways.

Participants in this study are Arabic-Hebrew-English (AHE) university student trilinguals in Israel. This population expands upon previously studied samples in three aspects – the specific language combination, the ubiquity of trilingualism, and the level of proficiency. In most research conducted on L3 processing, the linguistic background included L1 or L2 (or even both) that are typologically similar to L3, and all three languages often belonged to the same language family (often Indo-European, Ecke, 2015). In contrast, the current study focuses on trilinguals whose background languages (L1-Arabic and L2-Hebrew) are Semitic, and whose L3 (English) is Indo-European. Moreover, each of the three languages is written in a different script, such that when reading English there is no orthographic overlap with either the L1 or the L2. Thus, in the current study, the language typology factor is neutralized since both L1 and L2 are typologically distant from L3.

Participants in the current study are also recruited from a large population of trilingual speakers. Many previous studies of trilingualism have focused on individuals who have self-selected to become multilinguals by studying additional languages (e.g., Lindqvist, 2009; Ecke and Hall, 2013). However, the current study extends the literature to test individuals who have become trilingual due to their social-educational context. All native Arabic speakers in Israel study both Hebrew (which is also the majority societal language) and English (as a foreign language) from early elementary school (age 8–9). Research with these learners is important, because it allows us to test the generalizability of previous findings in wider populations. Recently, several studies have examined non-self-selected individuals, by comparing monolingual and bilingual learners acquiring an additional language (Fallah et al., 2016; Westergaard et al., 2017; Hopp, 2019; Lorenz et al., 2019), but these all tested children who were at relatively early stages of L3 acquisition.

Given the socio-educational system in Israel described above, native Arabic speaking university students are moderately proficient in both the L2 and the L3 (Prior et al., 2017). Specifically, at the time of testing, participants are partially immersed in the L2, in which they are conducting their studies, and are using L3 on a daily basis (see participant description below). Much previous theoretical interest has focused on early L3 acquisition, to identify the source of transfer in the initial state of learning (Rothman et al., 2019). Accordingly, in a recent systematic review of L3 learning, Puig-Mayenco et al. (2020) identified 40% of studies focusing on beginners, and the remainder as testing “post-beginners,” but they acknowledge that this is a very wide category. An examination of the studies included in the review shows that a much lower percent actually tested individuals who had been using the L3 for an extended period of time (over 10 years in the current study). Here, however, we chose to investigate intermediate proficiency trilinguals, who habitually use all three languages, to reach a better understanding of how cross-language influences continue to impact L3 processing beyond the initial stages of acquisition.

The current study also differs from previous research in our approach to selecting language materials. Most previous studies identified one or two syntactic structures, that either differed in the overlap with the L1 and L2 of a single group of participants (e.g., Hopp, 2019), or they included two groups with different L1/L2 constellations (termed Mirror-Image groups by Puig-Mayenco et al., 2020), and focused on a single structure (e.g., Falk and Bardel, 2011; Cabrelli-Amaro et al., 2015). In the current study, we adopt a different approach. The study includes a single group of trilingual participants, Arabic-Hebrew-English trilinguals, who are compared with a control group of monolingual native English speakers. Thus, the target language is English for all participants. For trilinguals, English is the L3, L1 is always Arabic and L2 is always Hebrew. We further focus exclusively on interference in morpho-syntactic processing, or “non-facilitative” transfer. Specifically, critical items are always ungrammatical in English the L3, but could be grammatical in L1, L2 or both (for a somewhat similar approach see Westergaard et al., 2017). Accordingly, we define 4 conditions of syntactic overlap: structures in L3 that mismatch both L1 and L2 (which share a similar structure), structures that mismatch either L1 or L2 (but are shared across English and the other language), and structures which are common across all 3 languages (deemed control). For each such condition, we identified at least 3 syntactic structures in English (and after pre-testing, at least 2 remained for full analysis). Note that this method by definition includes different syntactic structures in the 4 experimental conditions, and these may differ in their basic ease or difficulty of acquisition/processing in English. To control for these potential baseline differences, our study therefore includes a control group of monolingual native English speakers, whose performance across the experiment serves as the baseline to which trilingual performance is compared. Finally, the critical stimuli are always presented as ungrammatical sentences in English. Cross-language influence is probed due to the fact that the ungrammatical structure presented in English would be grammatical in participants’ L1, L2 or both. Our reasoning is that if there is indeed interfering cross-language influence from these languages, participants will find it more difficult to identify the English critical sentences as ungrammatical.

Finally, the current study includes both online and off-line measures of morpho-syntactic processing, by utilizing both recording of eye-movements during reading, and post-sentence grammaticality judgments. When overt decision tasks are used to study cross-language influences in L3 processing (e.g., Sanz et al., 2015; Slabakova and Garcia Mayo, 2015; Westergaard et al., 2017), participants normally wait to achieve a fairly high threshold level of certainty prior to responding. In contrast, the eye-movement record provides a window into the moment-by-moment processes underlying language comprehension (Dussias, 2010; Marinis, 2010; Sedivy, 2010). Recording eye-movements during reading provides a millisecond-precise report of the readers’ immediate syntactic processing. It also provides an extremely rich data set, and may be used to determine when (e.g., during the first or second pass through a sentence) and where exactly in a sentence processing difficulty occurs, as well as how the reader deals with such difficulty (e.g., by rereading/fixating for longer durations/regressive saccades to an earlier point in the sentence) (Conklin and Pellicer-Sánchez, 2016).

In the current study, we combined recording of eye-movements during reading L3 sentences, with a post-sentence judgment of whether it was grammatically well formed in English. This allows us to investigate ongoing interference during processing, as well as more meta-linguistic processes of offline judgments. However, it is important to note that some previous research has demonstrated that incorporating grammaticality judgments invokes greater strategic processing as well as greater sensitivity to reading patterns during online reading (Godfroid and Winke, 2015; Keating and Jegerski, 2015). Thus, we acknowledge that some of the reading patterns identified in the current study might not be perfectly aligned with those evident during naturalistic reading, when readers are not simultaneously engaged in an additional task.

The current study aims to examine whether cross-language influences in L3 morpho-syntactic processing can be identified from both the L1 and the L2 when typological similarity is neutralized. We hypothesize that both L1 and L2 are potential sources for interference in L3 processing, as suggested by the theoretical stance of the Linguistic Proximity Model and the Scalpel Model. Accordingly, we predict significant interference from L1 and from L2 when there is a mismatch in syntactic structure with the L3. Further, we hypothesize that interference might be increased when L3 differs from both background languages, suggesting that the degree of structural mismatch can modulate cross-language influences.

A second aim of the current study is to test whether proficiency and/or order of acquisition modulate cross-language influences. In particular, we ask whether cross-language influence from the more dominant language L1 is expressed earlier in the time course of processing than is cross-language influence from the less proficient L2. Early and late eye movement measures may be revealing in this respect. Finally, the combination of online and offline measures employed in the current study will allow us to test whether the impact of cross-language influences on real-time processing difficulty is similar to that expressed in metalinguistic based judgments.

## Method

### Participants

Fifty-three Arabic-Hebrew-English trilinguals (39 females, mean age 20.6) who were first year bachelor’s degree students at the University of Haifa participated in the study. Previous research shows that this population is most proficient in L1, then in L2, and least proficient in L3 (Prior et al., 2017). This dominance profile was verified using objective and subjective proficiency measures in each language (see details below, and Table 1 for participant characteristics). Participants grew up in exclusively Arabic speaking homes and schools. They started formal instruction in Hebrew at age 8 (2nd grade), had some exposure to Hebrew as the majority language in Israel, and at the time of data collection were immersed in college classes in Hebrew. Participants started formal instruction in English at age 9 (3rd grade), and had limited exposure to the language through media (music, television, film). Participants had no history of neurological or psychiatric deficits, learning or language disability and had intact or corrected vision. Nine participants were later excluded for not matching the required criteria, such that the final set of trilinguals included 44 participants (36 females, mean age *M* = 20.59, *SD* = 1.46, range 19–27, Parental education *M* = 14.6 years, *SD* = 4.1). Participants were recruited through advertisements and received course-credit or payment for participation.

**TABLE 1:** Trilingual participant characteristic (*N* = 44).

	L1 (Arabic)	L2 (Hebrew)	L3 (English)
Age began study	N/A	8.27 (1.67)	8.50 (1.22)
Current exposure (0–10 scale)*	7.17 (2.08)^a^	5.18 (1.84)^b^	3.55 (2.12)^c^
Self-rated Proficiency (0–10 scale)*	9.73 (0.49)^a^	8.02 (1.23)^b^	6.30 (1.53)^c^
Semantic Fluency Task*	23.20 (5.94)^a^	13.36 (6.92)^b^	11.86 (5.08)^b^
Phonemic Fluency Task^1^**	17.16 (5.15)^a^	13.50 (5.66)^b^	15.45 (4.35)^c^

*P < 0.001; Means in the same row with different superscript letters differ from each other significantly. Specifically, there were no significant differences in age of acquisition and in semantic fluency between L2 and L3.

**P < 0.05; Ratings of proficiency and exposure were averaged across productive and receptive oral and written language use.

*^1^Different phonemes were used across languages, informed by previous research. However, norming data collected in our lab after data collection of the current study revealed that these were not well matched across languages, with the English phonemes generating more responses than the Hebrew ones, which explains why these scores do not align well with the participants’ expected language profile.*

In addition, 37 monolingual native English speakers, students at the University of Wisconsin in Madison, participated in this study (33 females, mean age *M* = 20.02, *SD* = 1.46, range 18–25). They were recruited as a control group for the experimental task in order to set the baseline performance accuracy and reading times across the interference conditions (see detailed description below). All participants gave informed consent to take part in the study. The study was approved by the University of Haifa Ethics Committee.

### Materials

*L3 (English) Sentence Processing Task*. Critical sentences were ungrammatical sentences in English that included a violation in one of four types of constructions: (1) Similar in Arabic and Hebrew, but different in English (Double mismatch, causing interference from both L1 and L2); (2) Similar in Hebrew and English, but different in Arabic (L1 mismatch, causing L1 interference); (3) Similar in Arabic and English, but different in Hebrew (L2 mismatch, causing L2 interference); and (4) Similar in Arabic, Hebrew, and English (control, no interference). Examples for these conditions are presented in Table 2 (see Supplementary Materials 1 for full materials). For each condition, we identified 3 potential structures (see Supplementary Materials 2, for further examples and explanations), and constructed 5 sentences for each structure for a total of 15 sentences per condition^1^. Critical sentences were constructed by considering that reliance on syntactic rules of Arabic, Hebrew, or both, may lead to an error in judging the grammaticality of English sentences. If translated word by word, the ungrammatical sentences presented in English would be grammatical in one or both of the background languages (L1 and L2), depending on the condition. For instance, the English sentence “I am planning to buy dog^∗^ for my son’s tenth birthday” is ungrammatical in English because the indefinite article is omitted. However, the participants might find it difficult to detect this violation, because if directly translated into either Arabic or Hebrew, it would be grammatical, since neither language has indefinite articles. To deal with the diglossic nature of Arabic (Saiegh-Haddad and Henkin-Roitfarb, 2014), we only selected structures that are shared between the spoken and the written variants of Arabic.

**TABLE 2:** Examples of experimental materials: selected syntactic structures from the different mismatch conditions in Arabic, Hebrew, and English.

Condition	Construction	L3 (English)	L1 (Arabic)	L2 (Hebrew)
L1 mismatch/L2 similar	Possessive Marking	My classmate Adam always copies *Sara homework and the teacher never knows. [Sara’s homework]	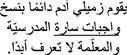	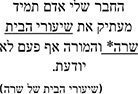
	Definite article omission in the superlative form	The coach likes Fadi because he is *fastest player in our team. [the fastest]	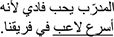	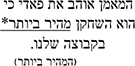
L1 similar/L2 mismatch	Comparative Form	My sister’s hair is *more long than my hair which is really short. [longer]	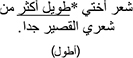	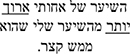
	Superlative Form	Everyone knows that I’m the *most rich in this neighborhood. [richest]	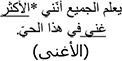	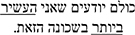
Double Mismatch: L1 mismatch/L2 mismatch	First person prodrop	Selena won’t talk to me even though *visited her last night. [I visited]	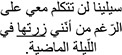	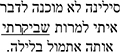
	Copula Omission	Ahmad won’t come with us because he *sick and tired today. [is sick]	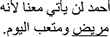	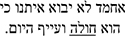
	Indefinite article omission	I am planning to buy *dog for my son’s tenth birthday. [a dog]	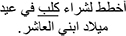	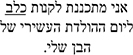
Control: No mismatch	Verb-time expression agreement	Yesterday, the students in my class *will go to Miami’s best beach. [went]	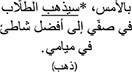	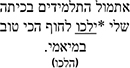
	Quantifier-noun agreement	Last week at the park, three *dog followed me, and I got scared. [dogs]	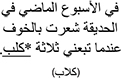	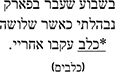

These 60 critical (ungrammatical) sentences were complemented by 60 (grammatical) filler sentences, constructed with no special constraint on cross-language influences. Both grammatical and ungrammatical sentences were simple active sentences, including high frequency vocabulary appropriate to participants’ proficiency level in English, as ascertained by a pre-test in which 31 Arabic-Hebrew-English trilinguals rated sentence grammaticality, and verified general familiarity with the vocabulary included in the sentences. Sentence length ranged from 10 to 14 words, and was matched across conditions [*F*(3,55) = 2.11, *p* = 0.109]. Critical words (the words at which the grammatical violation is evident) were preceded and followed by at least two content words.

To pre-test materials, 27 monolingual native English speakers, students at the University of Wisconsin in Madison rated the grammaticality of the sentences and identified the error in the ungrammatical sentences. These participants did not take part in the main experiment. The rating task was performed online, with each student rating 60 sentences, half of which were grammatical. These ratings, in concert with the performance of the native English speakers on the experimental task, were used to determine the baseline difficulty of the structures and to select the final set of structures, such that they were matched for difficulty across conditions for native English speakers (see “Results” section below).

*Language Proficiency Assessment.* Participants’ language profile was verified using both an objective verbal fluency task, and self-report measures derived from a detailed language history questionnaire, as detailed below.

*Verbal fluency tasks* (Gollan et al., 2002; Kavé, 2005). Participants were asked to produce in one minute as many words as possible within a given language for each of two semantic categories and each of two phonemes. In the semantic fluency task, three pairs of semantic categories (including one wide and one narrow category) were used and rotated randomly across the three languages for each participant: Animals and sports, fruits and occupations, and clothes and furniture (Gollan et al., 2002; Kavé, 2005). In the phonemic fluency task, a different pair of phonemes was used in each language in the following order: [ع] and [r] were used in Arabic, [b] and [ʃ] were used in Hebrew, [α] and [f] were used in English.

The order of languages was held constant across participants, so that both tasks (phonemic and semantic fluency tasks) were first administered in Arabic, then in Hebrew, and finally in English. The order of the tasks was counterbalanced, such that participants were randomly assigned to either complete the phonemic fluency task first and the semantic fluency task second, or vice versa. However, the same order was administered in the three languages for each participant (e.g., if in Arabic the semantic task was administered first, and the phonemic task was second, the same order was retained same in Hebrew and in English).

*Language History Questionnaire.* Participants completed an Arabic translation of the Language Experience and Proficiency Questionnaire (a modified version of the LEAP-Q, Marian et al., 2007) in which they provided self-ratings of language use, language exposure, and language proficiency (across speaking, understanding spoken language, and reading) in all acquired languages.

### Procedure

Arabic-Hebrew-English trilinguals performed the experimental task, in addition to language proficiency tests at the University of Haifa in a single session. The tasks were administered in the following order: English Sentence Processing task (including eye movement recording and post sentence grammaticality judgment task), Semantic and Phonemic fluency tasks, and then LEAP-Q (Marian et al., 2007). The order of test administration was held constant, except that the order of the fluency tasks was randomized. The entire experimental session lasted approximately an hour and a half.

Native English monolinguals completed the experimental task at the University of Wisconsin in Madison. They completed the identical English Sentence Processing task in a similar setting in Madison, except that the proper names included in the stimuli were English names and not Arabic (e.g., while the names “Ahmad” and “Yasmine” were used in the stimuli of the experimental group, the names “David” and “Jasmine” were used for the English speakers). They filled out a screening form to verify that inclusion criteria were met (monolingual speakers with no history of neurological or psychiatric disorder, learning or language disability and intact or corrected vision).

*English Sentence Processing Task.* Eye movements were recorded using an Eye Link 1000 eye tracker, which was tower-mounted in Haifa and desktop-mounted in Madison. Data were recorded monocularly from the pupil of the right eye at a sampling rate of 1,000 Hz. Chin and forehead rests were used to minimize head movement. Prior to the beginning of the experimental task, the eye-tracker was calibrated for each participant using a nine-point calibration grid, followed by a validation check. Then, the participants were presented with written instructions on the screen in their native language (Arabic or English). The instructions were followed by a practice block of 4 trials, and then by two experimental blocks of 60 trials each. The order of the sentences was set in the practice block and randomized in the experimental blocks.

Screen resolution was set at 1024 × 768 pixels, and sentences were presented in black Courier New 14-point font on a white background. Sentences were left justified, and before each sentence, a one-point calibration check on the left side of the screen was conducted to ensure that participants consistently began reading the sentences at the leftmost point. Trials were terminated when participants fixated a gaze-contingent box at the right bottom corner of the screen when they finished reading each sentence. Following each sentence, a question mark appeared in the middle of the screen and participants provided their grammaticality judgment by button press (right key for a grammatically correct sentence, left key for a grammatically incorrect sentence). Participants were instructed to use only grammaticality as the basis for their judgment, and were also instructed to respond as quickly and as accurately as possible. Feedback (smiley face/sad face on the screen) was provided in the practice block, but no feedback was given in the experimental blocks.

Participants were given a short break between the experimental blocks, and could also take a break at any point in the task between trials when necessary. The eye-tracking task took about 50 min to complete.

## Results

### Equating Baseline Performance – Subset Selection

As mentioned earlier, data from monolingual native English speakers was collected in order to gauge the processing difficulty of the various target structures, and to characterize the baseline complexity of processing each structure in the absence of any cross-language influence. Preliminary examination of the accuracy in the grammaticality judgment of the native English control group revealed, however, differences across experimental conditions [*F*(3, 108) = 27.18, *MSE* = 0.46, *p* < 0.001, η_*p*_^2^ = 0.43]. To achieve a clearer baseline for comparisons, four structures were eliminated, one from each condition: Adjective placement (Double mismatch condition), addition of a definite article prior to mass nouns (L1 mismatch condition), past progressive tense (L2 mismatch condition), and tense sequence (control condition). After eliminating these structures, accuracy of the native English speakers was equated across conditions [*F*(3,108) = 0.82, *MSE* = 0.025, *p* = 0.48, η_*p*_^2^ = 0.022]. The final set therefore included 9 constructions: 3 in the Double mismatch condition (11 items), 2 in the L1 mismatch condition (10 items), 2 in the L2 mismatch condition (10 items), and 2 in the control condition (10 items). Performance of the native English speakers on these remaining 9 constructions (see Table 3) was considered the baseline of performance in online and offline measures against which the performance of the AHE trilinguals was examined.

**TABLE 3:** List of remaining syntactic categories with different degrees of overlap in Arabic, Hebrew, and English.

	Cross language influence conditions			
	
	Double mismatch (interference from both L1 and L2)	L1 mismatch (interference only from L1)	L2 mismatch (interference only from L2)	Control condition (no interference)
Structures	First person pro-drop; Copula omission; Indefinite article omission	Possessive marking; Definite article omission in the superlative form	Comparative form; Superlative form	Verb-time expression agreement; Quantifier-noun agreement

### Analyses Approach and Model Structure

Reading measures were analyzed for the target word in each sentence, defined as the point in the sentence at which the grammatical violation became apparent. Thus, in the case where an obligatory constituent was omitted from the sentence to create the violation the following word was defined as the target word (in the sentence “I am planning to buy ^∗^dog for my son’s tenth birthday,” the word “dog” was defined as the target word). In cases where an incorrect form was used, it was defined as the target word (in the sentence “Everyone knows that I’m the ^∗^most rich in this neighborhood” the word “rich” was defined as the target word).

Grammaticality judgment accuracy, as well as 6 measures from the eye tracking record (First Fixation Duration, Gaze Duration, Total Time, Skipping, Regressions Out, Regressions In) were analyzed using linear mixed effect models, as these models allow one to simultaneously account for variance related to participants and to items. Grammaticality Judgments, Skipping and Regressions (In and Out) were analyzed following a binomial distribution (i.e., mixed logistic regression). Duration measures were log transformed to reduce skew in the distribution, as these transformations improved normality more than the inverse transformation. Within each measure, we first identified significant control variables, which were retained in subsequent models. Specifically, we used the *buildmer* function in the *buildmer* package (v. 1.3, Voeten, 2019) in R (version 4.0.3, R Core Team, 2020), which uses the (g)lmer function from the lme4 package (v 1.1.-21, Bates et al., 2015), to fit a model including all (normalized) control variables (participants’ age, target length, target frequency, sentence length in characters, average frequency of the words in the sentence, and averaged length of the words in the sentence). Using backward stepwise elimination, the *buildmer* function calculates p-values for all fixed effects based on Satterthwaite degrees of freedom using the *lmerTest* package (v. 3.1-0, Kuznetsova et al., 2017), or the Wald degrees of freedom for binomial distribution.

Once control variables were identified for each measure, we compared an additive model including the effects of Group and Mismatch Type (Model 1) against an interactive model including in addition the interaction between Group and Mismatch Type (Model 2) using Log Likelihood Ratio Test. The factors of interest were coded using treatment/dummy coding, such that for the effect of Group, Arabic-Hebrew-English (AHE) trilinguals were set as the reference against which native English (NE) speakers were compared. Similarly, for the effect of Mismatch Type, Control sentences were set as the reference against which L1 Mismatch, L2 Mismatch, and Double Mismatch sentences were compared. The random structure included by-participant and by-item intercepts, as well as by-participant slope for Mismatch Type and by-item slope for Group. In case of convergence failure, the random structure was simplified following the guidelines provided by Poort and Rodd (2019, removing correlations, removing slope with lowest variance while reintroducing correlations; removing correlations; removing the other slope). To probe interactive effects and conduct pairwise comparisons, we used the *testInteractions* function from the *phia* package (v.0.2-1, De Rosario-Martinez, 2015) with Bonferroni adjustments for multiple comparisons. Estimated means and standard errors (SE) were obtained via the *emmeans* package (v.1.5. 2-1, Lenth, 2020) the full R script of the analyses can be found in the Supplementary Materials.

### Analyses

Table 4 provides observed mean performance for each measure as a function of Group and Mismatch Type.

**TABLE 4:** Observed mean performance (SE) as a function of Group and Mismatch Type.

Group	Mismatch type	Measure						
		
		Gram. judgment	FFD	GD	TT	Skipping rate	Regressions out	Regressions in
AHE	Control	0.62 (0.03)	288 (7)	499 (20)	1058 (43)	0.03 (0.01)	0.26 (0.02)	0.56 (0.02)
	L1 Mismatch	0.44 (0.02)	281 (7)	600 (24)	1285 (52)	0.02 (0.01)	0.32 (0.02)	0.53 (0.02)
	L2 Mismatch	0.36 (0.02)	291 (8)	394 (14)	883 (40)	0.05 (0.01)	0.19 (0.02)	0.54 (0.02)
	Double Mismatch	0.56 (0.02)	286 (7)	479 (19)	1088 (41)	0.04 (0.01)	0.26 (0.02)	0.61 (0.02)
NE	Control	0.81 (0.02)	263 (6)	307 (10)	431 (15)	0.21 (0.02)	0.18 (0.03)	0.36 (0.03)
	L1 Mismatch	0.79 (0.02)	248 (5)	288 (9)	445 (15)	0.13 (0.02)	0.31 (0.03)	0.39 (0.03)
	L2 Mismatch	0.83 (0.02)	239 (5)	257 (7)	321 (12)	0.24 (0.02)	0.22 (0.03)	0.24 (0.03)
	Double Mismatch	0.77 (0.02)	259 (6)	302 (9)	424 (13)	0.21 (0.02)	0.18 (0.02)	0.40 (0.03)

*FFD, first fixation durations; GD, gaze durations; TT, total reading times.*

*SE calculated over all data points taking into account the presence of within-participant variables following (Morey, 2008). To this end, we used the function described by Change, W. http://www.cookbook-r.com/Graphs/Plotting_means_and_error_bars_(ggplot2)*

**FIGURE 1:**
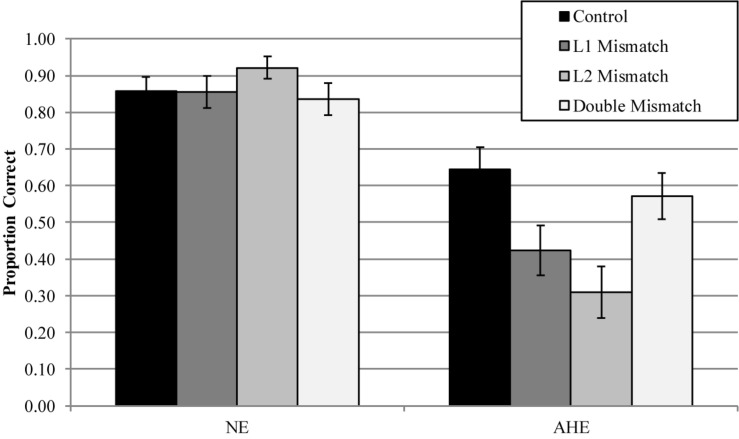
Estimated proportion correct in the grammaticality judgment for the effect of mismatch type as a function of group. Error bars represent SE.

*Grammaticality Judgment.* In the Grammaticality Judgment measure, model comparisons revealed that the interactive model (M2) improved the fit over the additive model [χ^2^ (3) = 13.75, *p* = 0.003]. Examination of model summary (see Table 6) revealed an interaction between Group and the difference between L2 Mismatch and Control, as well as a marginal interaction of Group with the difference between L1 Mismatch and Control. As seen in Figure 1, and supported by the pairwise comparisons with Bonferroni corrections for multiple comparisons (Table 7), the difference between L2 Mismatch and Control was larger for AHE [*b* = 0.80, χ^2^ (1) = 12.93, *p* = 0.004] than for NE [*b* = 0.34, χ^2^ (1) = 1.82, *p* = 1.00]. Further, the difference between L1 Mismatch and control was marginally significant for AHE [*b* = 0.71, χ^2^ (1) = 7.57, *p* = 0.071] but not for NE [*b* = 0.50, χ^2^ (1) = 0.00, *p* = 1.00]. Recall that the accuracy levels of the NE in the grammaticality judgment task was used to select the subset of constructions on which to examine the performance of AHE. Thus, it is not surprising that there are no differences across conditions in the NE group.

*First Fixation Duration.* In the FFD measure, model comparisons revealed that the interactive model (M2) did not improve the fit over the additive model [χ^2^ (3) = 4.53, *p* = 0.21]. Examination of model summary (Table 5) revealed a significant effect of Group, such that NE speakers had shorter FFD (*M* = 239, 95% CI [229, 249]) compared to AHE (*M* = 260, 95% CI [250, 271]).

**TABLE 5:** LME models predicting reading times (FFD, GD, TT).

	FFD			GD			TT		
			
Fixed effects	*b*	*SE*	*t*	*b*	*SE*	*t*	*b*	*SE*	*t*
(intercept)	5.58	0.03	196.64***	5.98	0.07	85.70***	6.78	0.07	96.16***
Group (NE)	−0.09	0.03	−2.99**	−0.34	0.10	−3.60***	−0.92	0.09	−10.53***
Mismatch (L1)	−0.02	0.03	−0.54	0.16	0.09	1.90^•^	0.00	0.06	−0.07
Mismatch (L2)	−0.04	0.03	−1.45	−0.12	0.09	−1.40	−0.22	0.07	−3.35**
Mismatch (Double)	0.00	0.03	0.08	−0.04	0.08	−0.45	−0.04	0.06	−0.61
Group(NE):Mismatch(L1)	–	–	–	−0.27	0.11	−2.35*	–	–	–
Group(NE): Mismatch (L2)	–	–	–	0.01	0.12	0.06	–	–	–
Group(NE): Mismatch (Double)	–	–	–	0.01	0.11	0.08	–	–	–
**Control variables**									
Participant’s age	–	–	–	–	–	–	−0.08	0.04	−2.17*
Target length	−0.03	0.01	−2.79**	0.07	0.01	5.08***	0.10	0.02	4.11***
Target frequency	−	−	−	−	−	−	−0.08	0.02	−3.39**

**Random effects**	**Variance**	**SD**	**Variance**	**SD**	**Variance**	**SD**			

Item (intercept)	0.00	0.05	0.03	0.17	0.04	0.20			
Group (NE)	0.00	0.04	0.00	0.06	0.06	0.25			
Group (AHE)	–	–	0.03	0.17	–	–			
Participant (intercept)	0.02	0.15	0.00	0.00	0.11	0.33			
Mismatch Type (L1)	0.01	0.12	0.04	0.21	0.02	0.12			
Mismatch Type (L2)	0.02	0.15	0.03	0.16	0.05	0.22			
Mismatch Type (Double)	0.01	0.07	0.04	0.19	0.02	0.13			
Mismatch Type (Control)	–	–	0.06	0.24	–	–			
Residual	0.13	0.36	0.22	0.47	0.30	0.55			

*Effect sizes (bs), standard errors (SEs), and *t*-values.*

FFD, first fixation durations; GD, gaze durations; TT, total reading times.

***p < 0.001; **p < 0.01; *p < 0.05; ^•^marginal.

**TABLE 6:** LME models predicting grammaticality judgment, skipping rate, regressions in, and regressions out.

	Gram judgment			Skipping rate			Regression in			Regression out		
				
Fixed effects	*b*	*SE*	*z*	*b*	*SE*	*z*	*b*	*SE*	*z*	*b*	*SE*	*z*
(intercept)	0.59	0.26	2.25*	−3.78	0.27	−13.80***	0.30	0.18	1.61	−1.29	0.20	−6.43***
Group (NE)	1.19	0.39	3.04**	2.07	0.25	8.23***	−0.94	0.27	−3.37***	−0.24	0.21	−1.12
Mismatch (L1)	−0.89	0.32	−2.75**	−0.32	0.30	−1.05	−0.17	0.18	−0.93	0.52	0.22	2.32*
Mismatch (L2)	−1.39	0.38	−3.59***	0.03	0.24	0.16	−0.09	0.19	−0.48	−0.30	0.24	−1.22
Mismatch (Double)	−0.30	0.32	−0.92	0.17	0.25	0.69	0.23	0.18	1.30	−0.03	0.22	−0.16
Group(NE): Mismatch (L1)	0.87	0.47	1.84^•^	–	–	–	0.31	0.27	1.16	–	–	–
Group(NE): Mismatch (L2)	2.05	0.58	3.50***	–	–	–	−0.6	0.30	−1.98*	–	–	–
Group(NE): Mismatch (Double)	0.13	0.47	0.28	–	–	–	−0.06	0.26	−0.23	–	–	–
**Control variables**												
Participant’s Age	0.06	0.12	0.55	−0.12	0.10	−1.2	–	–	–	–	–	–
Target Length	–	–	–	−0.67	0.10	−6.54***	–	–	–	–	–	–
Target Frequency	–	–	–	–	–	–	–	–	–	–	–	–

**Random effects**	**Variance**	**SD**	**Variance**	**SD**	**Variance**	**SD**	**Variance**	**SD**				

Item (intercept)	0.36	0.60	0.36	0.60	0.05	0.23	0.19	0.44				
Group (NE)	0.69	0.83	0.29	0.54	0.10	0.32	0.44	0.66				
Group (AHE)	–	–	–	–	–	–	–	–				
Participant (intercept)	0.74	0.86	0.33	0.58	0.81	0.90	0.47	0.69				
Mismatch Type (L1)	0.38	0.62	0.43	0.65	0.00	0.08	0.12	0.35				
Mismatch Type (L2)	2.16	1.47	0.05	0.22	0.20	0.44	0.32	0.56				
Mismatch Type (Double)	0.63	0.79	0.21	0.45	0.00	0.07	0.01	0.11				
Mismatch Type (Control)	–	–	–	–	–	–	–	–				

Effect sizes (bs), standard errors (SEs), and z-values.

***p < 0.001; **p < 0.01; *p < 0.05; ^•^marginal.

**TABLE 7:** Summary of pairwise comparisons with Bonferroni corrections for multiple comparisons as a function of Mismatch Type and Group.

		Grammaticality Judgment			Gaze Duration			Regression In		
				
Comparison		*b*	χ^2^	*p*	*b*	χ^2^	*p*	*b*	χ^2^	*p*
AHE	Control vs. L1 Mis.	0.71	7.57	0.07	−0.16	3.60	0.69	0.54	0.88	1.00
	Control vs. L2 Mis.	0.80	12.93	0.004**	0.12	1.95	1.00	0.52	0.24	1.00
	Control vs. Double Mis.	0.58	0.85	1.00	0.04	0.20	1.00	0.44	1.71	1.00
	L1 Mis. vs. L2 Mis.	0.62	1.71	1.00	0.28	10.90	0.012*	0.48	0.15	1.00
	L1 Mis. vs. Double Mis.	0.36	3.68	0.66	0.20	5.99	0.17	0.40	5.18	0.27
	L2 Mis. vs. Double Mis.	0.75	7.61	0.07^•^	0.08	1.00	1.00	0.58	3.01	1.00
NE	Control vs. L1 Mis.	0.50	0.00	1.00	0.10	3.78	0.62	0.46	0.37	1.00
	Control vs. L2 Mis.	0.34	1.82	1.00	0.11	4.26	0.47	0.67	7.05	0.095^•^
	Control vs. Double Mis.	0.54	0.15	1.00	0.03	0.31	1.00	0.46	0.56	1.00
	L1 Mis. vs. L2 Mis.	0.34	1.95	1.00	0.01	0.03	1.00	0.70	10.72	0.01*
	L1 Mis. vs. Double Mis.	0.54	0.14	1.00	−0.08	2.40	1.00	0.49	0.02	1.00
	L2 Mis. vs. Double Mis.	0.30	2.76	1.00	0.08	2.71	1.00	0.71	11.74	0.007**

***p < 0.001; **p < 0.01; *p < 0.05; ^•^marginal.

**FIGURE 2:**
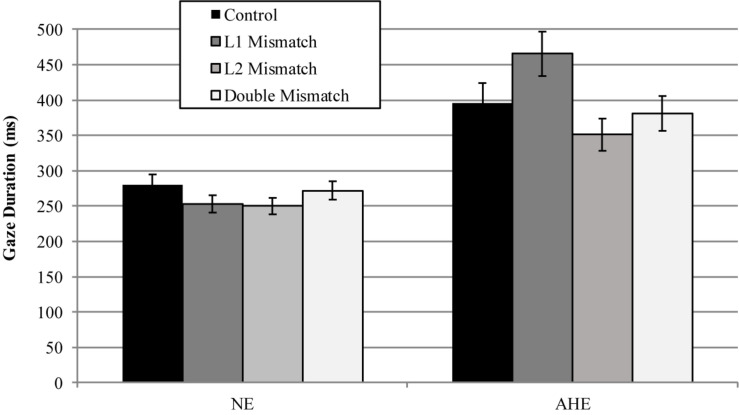
Estimated gaze durations for the effect of mismatch type as a function of group. error bars represent SE.

*Gaze Duration.* In the GD measure, model comparisons revealed that the interactive model (M2) improved the fit over the additive model [χ^2^ (3) = 8.74, *p* = 0.03]. Examination of model summary (see Table 5) revealed that the difference between L1 Mismatch sentences and Controls was modulated by Group (see Figure 2). Pairwise comparisons with Bonferroni corrections for multiple comparisons (Table 7) revealed however, that only the difference between L1 Mismatch and L2 Mismatch in the AHE reached significance [*b* = 0.28, χ^2^ (1) = 10.90, *p* = 0.011].

*Total Time*. In the TT measure, model comparisons revealed that the interactive model (M2) did not improve the fit over the additive model [χ^2^ (3) = 1.58, *p* = 0.66]. Examination of model summary (Table 5) revealed a significant effect of Group, such that NE speakers had shorter Total reading times (*M* = 330, 95% CI [294, 371]) compared to AHE (*M* = 827, 95% CI [739, 927]). In addition, total reading times for the target word in the L2 Mismatch condition were shorter (*M* = 476, 95% CI [426, 533]) than for target words in the control No Mismatch condition (*M* = 593, 95% CI [531, 662]).

*Skipping.* When examining Skipping Rates, model comparisons revealed that the interactive model (M2) did not improve the fit over the additive model [χ^2^ (3) = 0.43, *p* = 0.93]. Examination of model summary (Table 6) revealed an effect of Group, such that NE speakers skipped the target word more often (*M* = 0.15, 95% CI [0.12, 0.19]) than AHE trilinguals (*M* = 0.02, 95% CI [0.01, 0.03]).

*Regressions Out.* For the Regressions Out, model comparisons revealed that the interactive model (M2) did not improve the fit over the additive model [χ^2^ (3) = 4.69, *p* = 0.20]. Examination of model summary (Table 6) revealed more regression out of targets in the L1 Mismatch condition (*M* = 0.30, 95% CI [0.23, 0.37]) relative to Control (*M* = 0.20, 95% CI [0.15, 0.26]), but this effect was not modulated by Group.

**FIGURE 3:**
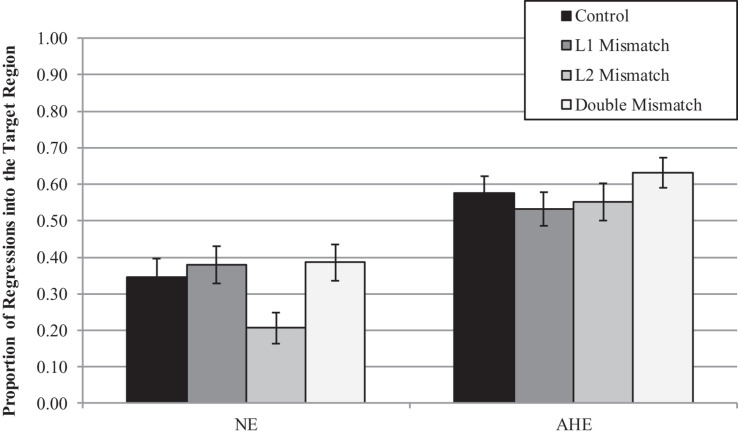
Estimated proportions of regressions into the target region as a function of mismatch type and group. Error bars represent SE.

*Regression In.* When examining Regression Into the target area, model comparisons revealed that the interactive model (M2) improved the fit over the additive model [χ^2^ (3) = 9.05, *p* = 0.03]. Examination of model summary (Table 6) revealed that the difference between L2 Mismatch sentences and Controls was modulated by Group. As seen in Figure 3, and supported by pairwise comparisons with Bonferroni corrections (Table 7), whereas NE control participants regressed less into targets of sentences in the L2 Mismatch condition relative to the other conditions, this difference was not present for AHE trilinguals.

## Discussion

The current study examined L1 and L2 as potential sources of cross-language influences during L3 processing when typological similarity is neutralized. In accordance with our hypotheses, we observed interference from both background languages in L3 processing. L1 interference was observed in earlier online measures of processing than L2 interference, and both were also observed in offline judgments. Surprisingly, however, whereas structural mismatch with a single background language (either the L1 or the L2) resulted in significant interference, structural mismatch with both background languages did not lead to significant interference. We address each of these findings below.

The current findings demonstrate that structural mismatch between the L3 and either the L1 or the L2 of trilingual speakers resulted in significant interference. Specifically, participants were less accurate at identifying ungrammatical sentences in English when the corresponding structure was grammatical in the L2, and marginally so when the structure was grammatical in the L1. Further, participants had longer gaze durations in the critical target area when reading L1 mismatch ungrammatical sentences. When reading L2 mismatch ungrammatical sentences, first pass reading times were not affected but participants made more regressions back into the target area than would be expected based on the performance of native English speakers who do not experience cross-language influence. These findings indicate the presence of interference from both the L1 and the L2 of trilingual speakers, and align with the theoretical stance put forth by the Linguistic Proximity Model (Westergaard et al., 2017) and Scalpel Model (Slabakova, 2017). According to these models, cross-language influence is determined on a structure by structure basis, and neither background language has a privileged role in supplying cross-language influences.

Extending previous literature, the current study further reveals time-course differences in the operation of cross-language influence from L1 and L2. Specifically, interference from L1 (Arabic) was evident early on, in first pass reading measures. Gaze durations to target words in L1 mismatch structures were longer than gaze durations to target words in sentences with control structures, for AHE trilinguals but not for monolingual English speakers. Sensitivity to L1 interference was not apparent in later reading measures, such as total time and regressions in, but was marginally significant in the offline grammaticality judgment measure. Interference from L2 (Hebrew), however, was not evident in the early measures of online processing (first fixation durations, gaze durations, skipping or regression out), but was apparent in the second pass reading measure of regressions into the target region. In comparison to the monolingual English speakers, who exhibited reduced regressions into the target region in sentences in the L2 mismatch condition, the AHE trilinguals exhibited equal rates of regressions into the target region in the L2 mismatch and Control conditions. We interpret this pattern as indicating that the structures in the L2 mismatch condition were easier for native English speakers than those in the Control condition, given that these are different structures, but critically that AHE trilinguals did not show this expected facilitation due to interference from the L2. The offline measure supports this interpretation, as the AHE trilinguals were much less accurate in their grammaticality judgment decisions on sentences in the L2 mismatch condition compared to control sentences.

Thus, the answer to the question which of a trilinguals’ background languages exerts stronger cross-language influence during L3 processing appears to depend on the measure. Specifically, L1 interference was evident earlier in processing, but L2 interference was stronger in the offline metalinguistic measure. One possible explanation for this pattern is that because trilinguals were sensitive to L1 interference already during first pass reading, they were more successful in resolving this interference by the time they performed the grammaticality judgment after completing reading the sentence. In contrast, because sensitivity to L2 interference emerged only later in sentence processing, in second pass reading measures, it was not yet resolved, and thus exerted a stronger influence on sentence final grammaticality judgments.

By adding sensitive measures of cross-language influence during online processing, we were able to identify a nuanced pattern of results. Specifically, although cross-language influence from the L1 was only marginal in the sentence-final grammaticality judgment, it was robust during the earlier measure of reading time. Further, the difference in timing between cross-language influences from the L1 and the L2 only emerged in the real-time online measures. Such divergence between real-time online measures and offline grammaticality judgments has been observed and influential in previous studies on L2 learning (e.g., McLaughlin et al., 2004; Tokowicz and MacWhinney, 2005). We therefore believe that incorporating similar measures of online processing to studies of cross-language influences in L3 processing is a fruitful avenue which might be useful in reconciling some of the conflicting findings in the extant literature. Nevertheless, it is important to note that our experimental design incorporated both eye-movement data and grammaticality judgments following each sentence, which could have influenced natural reading and activated greater metalinguistic awareness (Valdés Kroff et al., 2018). Therefore, future research should continue to investigate this issue by separating online and offline tasks in order to maintain cleaner measures.

We predicted the strongest degree of interference in structures that mismatch both the L1 and the L2, because the entirety of participants’ background linguistic knowledge conflicted with the L3 in these structures. However, not only did we not find stronger interference under these conditions, but in fact interference was not significant for these structures across the different measures – either online or offline. We propose that these structures may have been particularly salient for the AHE trilinguals tested in the current study, for one of two possible reasons. First, it is possible that when an L3 learner encounters a structure that differs from both of her background languages this draws attention and emphasizes the need to relearn a morpho-syntactic feature of the L3 (Schmidt, 2012). Thus, the morpho-syntactic structures in this category can be considered as being unique to the trilinguals’ L3, because they are unattested in either the L1 or the L2, and may thus gain particular salience to learners (Tokowicz and MacWhinney, 2005; Tolentino and Tokowicz, 2014). As a result, speakers may become more aware of the potential error on such structures and thus monitor their performance on this feature more closely. Second, it is possible that these specific morpho-syntactic features of English as an L3 are explicitly highlighted during instruction because of the mismatch with participants’ background languages. These options are not mutually exclusive, but future research might be able to distinguish among them by testing less proficient trilinguals from the same population. If the driving force is explicit instruction, less proficient trilinguals would demonstrate relative ease with processing such Double mismatch structures just as high proficiency trilinguals do. If, however, this facility in processing arises slowly with growing L3 proficiency and meta linguistic knowledge, we would expect lower proficiency trilinguals to indeed show increased interference for the Double mismatch structures.

Extending previous studies of L3 learning, which have largely focused on the initial stages and on individuals who have self-selected to become multilinguals (e.g., Ecke and Hall, 2013), here we tested individuals who have become trilinguals due to their socio-educational context and are moderately proficient users of L3 English. Thus, the results of the current study carry the potential to be more generalizable to typical multilinguals in today’s global society (Kaushanskaya and Prior, 2015).

Further, our approach to selecting language stimuli differs from that adopted by most previous research. Namely, we identified a wider number of syntactic structures different from each other in their mismatch with participants’ L1 and L2. This allowed us to simultaneously measure cross-language influences from both background languages in a single group of participants, which has the advantage of greatly reducing potential differences (in language learning background, proficiency in L1/L2) that might arise in between-participant comparisons, even in “Mirror Group” designs (Giancaspro et al., 2015). However, this approach has the inevitable result that the experimental conditions included different syntactic structures, which introduces a different source of variability, such as potential baseline differences in sensitivity or salience of the selected structures. Even though some structures were eliminated so that accuracy of the native English speaking control group in the grammaticality judgment was equated across conditions, it is possible that some variability remained unaccounted for. Indeed, the pattern observed in the regressions-in measure for native English monolingual speakers suggests such baseline variability. Thus, the current results should be interpreted as complementary to those arising from other methodologies, to lead to a fuller nuanced understanding. Future research can explore alternative means of matching between structures, or directly compare the results of experiments using these different methodological approaches.

Finally, cross-language influences can manifest as either facilitation or interference, the latter of which was the focus of the current study. Importantly, it is currently unclear whether facilitation and interference effects are symmetrical, and whether they are similarly easy to detect. Indeed, often the direction of influence is determined by the type of manipulation examined in a particular study, and specifically by how researchers define the baseline condition. Thus, in the lexical domain for instance, items that are non-cognates typically serve as controls, against which overlap in form and meaning (cognates) results in facilitation but overlap in form (but not meaning) results in interference (at least in processing, but not in learning, e.g., Hirosh and Degani, (accepted); Elias and Degani, unpublished; Marecka et al., 2020). In the syntactic domain, some researchers have treated unique syntactic constructions as a baseline, such that when constructions that are similar across languages are compared to this baseline facilitation is expected, but when structures that differ across languages are compared to the unique baseline condition, interference is expected (e.g., Tokowicz and MacWhinney, 2005). The pattern of results, however, is more complex, as unique structures are sometimes experienced as especially difficult (Tokowicz and Warren, 2010). Somewhat analogously, in the current study we defined our baseline as constructions that are shared across all three languages, such that constructions that are not shared by (at least) one language will index interference. Our matching procedures were therefore conducted between the interference conditions and the selected baseline. Alternatively, one could have selected constructions unique to English as the baseline, against which constructions that also overlap with the L1, with the L2 or with both, will index facilitation. Future studies may be useful in examining whether facilitative and interfering cross-language influences operate similarly for bilingual and trilingual speakers.

## Conclusion

Overall, the findings of the current study suggest that the entire linguistic repertoire is activated when processing L3. These findings are consistent with the Linguistic Proximity Model (Westergaard et al., 2017) and the Scalpel model (Slabakova, 2017). Our results demonstrate that neither L1 nor L2 are the single default supplier for cross-language influence, and that all previously learned languages may shift and modulate the linguistic system. Going beyond typological proximity, the current study documents robust cross-language influences across languages that are typologically distinct. Finally, the current study sheds light on the difference between performance in online and offline measures, and how processing language in real-time differs from judgments that rely on meta-linguistic knowledge (Dussias, 2010; Sedivy, 2010). In addition to theoretical insights, the current study has important implications for L3 language instruction. Specifically, our results suggest that difficulties in L3 learning might not only be a result of interference from the L1 (Tajareh, 2015), but could also reflect cross-language influences from the L2. Thus, when scaffolding L3 learning, both L1 and L2 should be taken into account as influential background languages.

## Data Availability Statement

The raw data supporting the conclusions of this article will be made available by the authors, without undue reservation.

## Ethics Statement

The studies involving human participants were reviewed and approved by Ethics Committee of the Faculty of Education at the University of Haifa. The participants provided their written informed consent to participate in this study.

## Author Contributions

NA, TD, and AP designed the study, analyzed data, and wrote the manuscript. NA created stimuli and collected data. All authors contributed to the article and approved the submitted version.

## Conflict of Interest

The authors declare that the research was conducted in the absence of any commercial or financial relationships that could be construed as a potential conflict of interest.

## Supplementary Material

The Supplementary Material for this article can be found online at: https://osf.io/c625n/ (identifier: DOI: 10.17605/OSF.IO/C625N).
